# Performance Evaluation of SmartBrain: A Wearable PET System for Human Brain Imaging

**DOI:** 10.2967/jnumed.125.271350

**Published:** 2026-08

**Authors:** Han Liu, Wenkang Qu, Da Liang, Xin Yu, Yuejie Lin, Haoyu Zou, Siyuan Han, Zhijun Zhao, Ying Lin, Xiaoyin Zhang, Jinyong Tao, Wenbin Li, Huiping Zhao, Yibin Zhang, Gongning Luo, Ningyi Jiang, Qiyu Peng

**Affiliations:** 1 Institute of Biomedical Engineering, Shenzhen Bay Laboratory, Shenzhen, China;; 2 College of Artificial Intelligence, Harbin Institute of Technology, Harbin, China; and; 3 Department of Nuclear Medicine, Seventh Affiliated Hospital, Sun Yat-sen University, Shenzhen, China

**Keywords:** wearable PET, human brain imaging, NEMA, SmartBrain

## Abstract

Brain PET is a powerful imaging modality that directly reflects cerebral metabolic activity, making it an essential tool for human brain research. However, conventional brain PET typically requires subjects to remain stationary in a sitting or supine position during scanning. This study evaluated the performance of SmartBrain, a wearable brain PET system for real-time imaging. **Methods:** SmartBrain uses 192 detectors, with each detector consisting of a 6 × 6 lutetium–yttrium oxyorthosilicate crystal (3 × 3 × 5 mm^3^) array with a 3 × 3 silicon photomultiplier array. We evaluated the physical performance of SmartBrain in accordance with the National Electrical Manufacturers Association (NEMA) NU 2-2018 standard. In addition, we performed ^18^F-FDG imaging using a custom Hoffman brain phantom and a multilayer Derenzo phantom. Dynamic rat images and the ^18^F-FDG images from a healthy volunteer are presented. **Results:** Spatial resolution is 2.29 mm in the center of the field of view. The sensitivity was 720.2 cps/MBq. The peak noise-equivalent count rate was 4.67 kcps at 10.1 kBq/mL, and the scatter fraction was 29.5%. The NEMA image-quality contrast recovery coefficients ranged from 72.9% (10-mm sphere) to 89.4% (37-mm sphere), and background variability was 11.3% at a contrast ratio of 9.4:1. The time-of-flight resolution was 234 ps, and the energy resolution was 10.8%. SmartBrain showed that the main structures of the custom Hoffman brain phantom could be resolved and demonstrated the ability to separate rods as small as 1.7 mm. **Conclusion:** SmartBrain clearly demonstrated brain structures, confirming its suitability for clinical brain research. Moreover, as a wearable and mobile PET platform, it offers unique opportunities for naturalistic brain imaging and future clinical applications in the diagnosis and management of neurologic disorders.

## Visual Abstract

**Figure:**
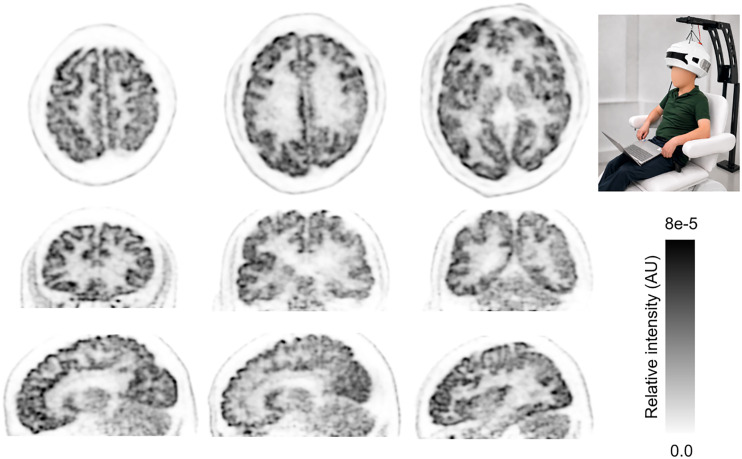

Human brain research is essential for understanding cognition, emotion, and behavior. Brain PET is a powerful imaging modality that directly reflects cerebral metabolic activity, making it an essential tool for human brain research (1,2). However, conventional brain PET typically requires subjects to remain stationary in a sitting or supine position during scanning, which poses limitations for certain patient populations, such as children and individuals with epilepsy or other neurologic disorders. Wearable brain PET systems aim to address these challenges by enabling brain metabolism studies in more natural or even dynamic conditions, thereby expanding the potential applications of brain PET in neuroscience and clinical research. Moreover, wearable brain PET systems are highly portable and require less space and are more cost-effective compared with conventional PET systems, which could allow this technology to be extended to a broader population.

Despite this potential, research on mobile or wearable brain PET systems remains at an early stage. In 2011, Yamamoto et al. developed a seated PET brain imaging system, known as PET-Hat, specifically designed for human studies (3). Subsequently, Tashima et al. introduced a similar seated PET system (Helmet-Chin PET) (4). However, both systems require stationary mounting platforms, limiting their use in portable imaging contexts. In 2017, Majewski et al. developed a semimobile brain PET system (AM-PET) by mounting the PET scanner on an externally movable support (5). In the same year, Xu et al. advanced this technology by creating Mind-tracker PET, a fully wearable brain PET system weighing 3.5 kg that operated without an external support structure (6). However, both AM-PET and Mind-tracker PET had limited axial fields of view (FOVs) (50 and 30 mm, respectively), restricting imaging to small regions of the brain. Moreover, these systems lacked time-of-flight (TOF) measurement capabilities (7).

We developed a wearable brain PET system (SmartBrain) capable of imaging in free movement. To verify whether it meets the basic standards of commercial PET systems, we evaluated SmartBrain’s performance using the National Electrical Manufacturers Association (NEMA) NU 2-2018 standard and compared it with the Discovery MI PET/CT system (DMI; GE HealthCare).

## MATERIALS AND METHODS

### System Parameters

**FIGURE 1.**
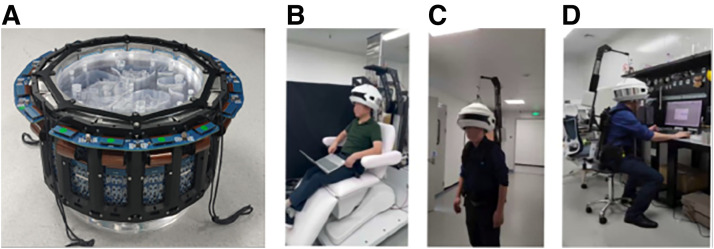
SmartBrain system. (A) System comprises detectors and silicon photomultipliers with custom Hoffman brain phantom. (B) Seated-position scanning. (C) Backpack system permitting data collection in ambulatory states. (D) Workstation scenario demonstrating natural seated use.

The SmartBrain system (Fig. 1) comprises a 16-sided polygonal ring (207.44-mm inner diameter, 121-mm axial FOV) with 192 detector modules arranged in 6 rings (32 per ring, 0.5-mm gaps). Each module comprises a 6 × 6 array of 3 × 3 × 5 mm^3^ lutetium–yttrium oxyorthosilicate crystals, optically coupled via optical adhesive to a 3 × 3 array of 6-mm silicon photomultipliers (onsemi).

A channel-reduction strategy decreases the total number of readout channels from 1728 to 432. Each detector module integrates a 3 × 3 silicon photomultiplier array. Four modules are combined in a 2 × 2 configuration, and signals are hierarchically multiplexed from 36 channels to 9 channels by encoding 3 rows, 3 columns, and 3 logical layers, with the fourth layer inferred from the others (8). To further reduce system weight, the 5-mm-thick crystals were used, at the cost of lower sensitivity and signal-to-noise ratio. Future integration of TOF is expected to improve the signal-to-noise ratio (9). The use of channel reduction and lightweight crystals resulted in an overall system weight of approximately 6 kg.

The system supports list-mode acquisition (energy window, 461–611 keV; coincidence window, 1 ns), with a decoding algorithm ensuring accurate event positioning. Data are time-stamped for flexible postprocessing, supporting TOF and dynamic imaging. Two mechanical support systems were developed: a wearable backpack and a suspension system (Fig. 1). Temperature is stabilized within 0.5 °C using external fans and air-cooled ducts.

### Performance Evaluation Using NEMA NU 2-2018

Physical performance, including spatial resolution, sensitivity, count rate performance, accuracy of corrections, image quality, energy and timing resolution, was evaluated in accordance with the NEMA NU 2-2018 standard (10).

#### Spatial Resolution

Spatial resolution was measured at 16 different locations, with 60-s acquisitions at each point (8 points at the center of the axial FOV [*Z* = 1/2], 8 points at one-eighth of the axial FOV from the end of the tomography [*Z =* 1/8]), using a 0.25-mm ^22^Na point source (0.4 MBq) embedded in a solid disk.

For the NEMA spatial resolution evaluation, the data were reconstructed using a 2-dimensional filtered backprojection algorithm without any correction, with a voxel size of 0.543 × 0.543 × 1.625 mm^3^ and a matrix size of 303 × 303 × 255. To meet the NEMA requirement that the voxel size be smaller than one-third of the full width at half maximum, the reconstructed images were resampled using trilinear interpolation to achieve an isotropic voxel size of 0.5 × 0.5 × 0.5 mm^3^. Axial, radial, and tangential resolutions were evaluated according to the NEMA NU 2-2018 standard.

In addition, to more thoroughly assess the intrinsic point-source resolution of the system, the central point-source data were also reconstructed using a maximum likelihood expectation–maximization algorithm with 1000 iterations. The reconstruction used an isotropic voxel size of 0.5 × 0.5 × 0.5 mm^3^, a matrix size of 361 × 361 × 243, and a point-spread function (PSF) model (1.625-mm gaussian kernel).

#### Sensitivity

Sensitivity measurements were performed using a NEMA PET sensitivity phantom consisting of 5 concentric aluminum sleeves, each 70 cm in length. A 70-cm polyethylene tube filled with 5.1 MBq of ^18^F-FDG solution was inserted into the aluminum sleeves, positioned both at the center of the transaxial FOV and 5 cm off-center. Ten 600-s acquisitions were conducted, successively removing the outermost sleeve to estimate attenuation-free sensitivity. The collected list-mode data were binned into sinograms using single-slice rebinning to estimate both total sensitivity and axial slice sensitivity.

#### Count Rate Performance

Count rate performance was evaluated using a custom-designed scatter phantom consisting of a polyethylene cylinder (length, 14 cm; diameter, 10 cm). Because of the geometric constraints of SmartBrain, the phantom’s geometry combined the hole configuration scaled from the NEMA NU 2-2018 human phantom with the overall diameter specified for the NEMA NU 4-2008 (11) monkey-size phantom, resulting in a radially offset line source positioned 22 mm from the center. The line source was initially loaded with 41.9 MBq of ^18^F-FDG at the first measurement start and 1.2 MBq at the last measurement start. In total, we scanned 34 frames with 60-s durations as the activity decayed. The count rate and scatter fraction calculations followed the alternative analysis with no randoms estimate, as specified in NEMA NU 2-2018 (section 4.4.2). System count rates were plotted alongside activity concentrations, including true, random, and scatter events; total counts, noise-equivalent count rate; and scatter fraction, all reported without correction.

#### Image Quality

We developed a brain-sized image quality phantom to replace the human torso, which had 6 spheres as the original phantom. A cylindric phantom with a liquid-filled diameter of 19 cm was filled with ^18^F-FDG at a background activity concentration of 4.1 kBq/mL. Six spheres (diameter range, 10–37 mm) were filled with an ^18^F-FDG solution at a sphere-to-background activity ratio of 9.4:1 to ensure sufficient counting statistics for the small custom phantom, although this may lead to elevated apparent contrast recovery coefficients compared with NEMA-recommended conditions. Measurement duration was 60 min. Contrast of hot spheres and background variability were measured according to the NEMA NU 2-2012 standard (12). To evaluate background variability, 12 circular regions of interest (10 mm) were placed on the background uniform region over 5 axial slices (60 regions of interest in total). Images were reconstructed using the ordered-subset expectation maximization (OSEM) algorithm for 12 iterations with 4 subsets. Only PSF modeling (2-mm gaussian kernel) and attenuation correction were applied. The voxel size was 1 × 1 × 1 mm^3^ and the shape matrix size was 217 × 217 × 120.

#### Energy and Timing Resolution

Energy and timing resolution were assessed using the same scaled-down NEMA scatter fraction phantom used in the count rate performance evaluation, with approximately 10.6 MBq of ^18^F-FDG. The central axis of the phantom coincided with the PET *Z*-axis, and the radioactive source was positioned using the TOF-free OSEM reconstructed image. For timing calibration, a 6-line source was positioned 50 mm from the center to perform system timing calibration. Each line source had an inner diameter of 0.8 mm and a length of 140 mm, with an injected activity of 10.2 MBq for ^18^F-FDG. Data were acquired for 20 min with an energy window of 461–611 keV and an initial coincidence timing window of ±5 ns. Timing calibration was iteratively performed after the lines of response selection until the change in timing resolution between iterations was less than 1 ps, after which a narrower timing window was applied. In accordance with the NEMA standard, lines of response of more than 20 mm from the radioactive source were excluded, and scatter and random contributions were estimated from the tail of the timing histogram (time bin, 10 ps).

### Phantom Studies

The multilayer Derenzo phantom used in this study was filled with an initial activity of 14.5 MBq of ^18^F-FDG and scanned for 70 min for imaging evaluations. The phantom (Supplemental Fig. 1, available at http://jnm.snmjournals.org) consists of 3 layers of rod sources with varying diameters. The phantom comprises 3 axial layers of rod sources located at 0–33 mm (1.5–2.0 mm, 0.1-mm increments), 47–67 mm (2.0–4.0 mm, 0.4-mm increments), and 72–121 mm (2.5–5.0 mm, 0.5-mm increments) within the PET axial FOV. The rods were arranged in a standard hexagonal pattern, allowing resolution comparisons across a range of feature sizes in a single acquisition. The phantom was positioned at the center of the FOV with a 5-mm offset. Images were reconstructed using the OSEM algorithm (12 iterations, 4 subsets) with PSF modeling (2-mm gaussian kernel), with and without TOF information. The voxel size was 0.5 × 0.5 × 0.5 mm^3^, and the shape matrix size was 217 × 217 × 217. Three experiments were performed; 1 TOF-enabled central placement is described here, and the other 2 (non-TOF, central and 5-cm off-center) are presented in the supplemental material.

Additionally, a custom-designed Hoffman brain phantom was prepared and filled with 24.0 MBq of ^18^F-FDG, followed by 15-min scan. The phantom had an outer diameter of 190 mm, a thickness of 146 mm, a liquid-filled diameter of 170 mm, and an axial length of 95 mm. The brain-structure insert consisted of 19 slices, each 5-mm thick, with a maximum diameter of 144 mm and the same axial length (95 mm) (Supplemental Fig. 2). In this design, the gray matter regions contained radioactivity, whereas the white matter and ventricular regions were nonradioactive. Images were reconstructed using the OSEM algorithm (12 iterations, 4 subsets) with random, attenuation, scatter correction, and PSF modeling (3-mm gaussian kernel) incorporating TOF information. Since the system is not equipped with CT, attenuation correction was performed using a uniform cylindric attenuation map with the same size as the phantom. The voxel size was 1 × 1 × 1 mm^3^, and the shape matrix size was 217 × 217 × 120.

### Human Study

A 43-y-old male patient with epilepsy participated in a brain ^18^F-FDG PET protocol approved by the medical ethics committee of the Seventh Affiliated Hospital of Sun Yat-sen University (KY-2024-354-02). Written informed consent was provided by the patient. His blood glucose level was 100.8 mg/dL, and the injected activity was 318.2 MBq.

A comparative evaluation was performed between the SmartBrain system and the DMI PET/CT scanner. First, an 8-min PET/CT scan was acquired on the DMI at approximately 60 min postinjection with the patient at rest. Images were reconstructed using OSEM (2 iterations, 17 subsets) with TOF and standard corrections (attenuation, scatter, randoms, normalization, calibration, dead time). The voxel size was 1.30208 × 1.30208 × 2.79 mm³, and the shape matrix size was 384 × 384 × 71.

Subsequently, a 60-min PET scan was performed using SmartBrain at 190 min postinjection (activity, ∼95.9 MBq). Images were reconstructed using OSEM (12 iterations, 4 subsets) with random, attenuation, and scatter corrections, as well as PSF modeling (3-mm gaussian kernel) incorporating TOF information. The reconstructed images contained 217 × 217 × 120 voxels with a voxel size of 1 × 1 × 1 mm³. CT images from the DMI system were used for attenuation correction.

## RESULTS

### Performance Evaluation Using NEMA NU 2-2018

**FIGURE 2.**
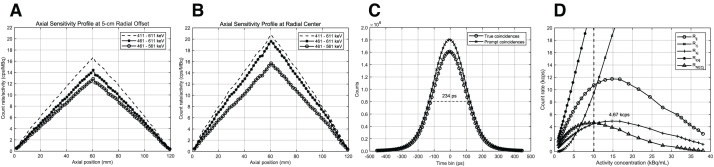
(A) Axial sensitivity profile with line source at 5-cm radial offset. (B) Axial sensitivity profile with line source at center of FOV. (C) TOF resolution with 10-ps time bins. (D) True coincidence (R_tj_), randoms (R_rj_), scatter events (R_sj_), prompt coincidences (R_totj_), and noise-equivalent count (R_NECj_) rates.

The mean 2-dimensional filtered backprojection spatial resolution at the center of the axial FOV was 2.29 mm (Table 1). The apparent Gibbs phenomenon observed at off-center positions was most likely attributable to limited angular sampling and crystal width. On this basis, the central point-source data with added background activity were further reconstructed using a maximum likelihood expectation–maximization algorithm with 1000 iterations, as previously described (13). Under these conditions, the estimated spatial resolution approached 1.7 mm, as illustrated in Supplemental Figures 3 and 4, and is consistent with the smallest visually resolvable rods observed in the Derenzo phantom experiment (Supplemental Fig. 5). The total system sensitivity at the center was 720.2 cps/MBq within the energy window of 461–611 keV (Fig. 2B). The measured average system TOF resolution was 234 ps in (Fig. 2C), and the energy resolution was 10.8% (full width at half maximum). The peak noise-equivalent count rate was 4.67 kcps at an activity concentration of 10.1 kBq/mL (Fig. 2D), and the scatter fraction was 29.5%. The NEMA image-quality contrast recovery coefficients varied from 72.9% (10-mm sphere) to 89.4%, and the background variability was 11.3% (12 iterations, 4 subsets) (Table 2).

**TABLE 1.** Intrinsic Spatial Resolution Measurements at 16 Locations

	Spatial Resolution (mm)			
Offset (cm)	Average	Radial	Tangential	Axial
*Z* = 1/2				
0	2.29	2.50	2.77	1.61
1	2.49	2.84	3.02	1.61
2	2.44	2.58	3.12	1.61
3	2.72	2.83	3.71	1.61
4	2.65	2.06	4.27	1.61
5	2.93	2.74	4.46	1.61
6	3.41	2.34	6.29	1.61
7	3.52	1.95	7.00	1.61
*Z* = 1/8				
0	2.87	3.25	2.27	3.08
1	3.24	3.53	3.13	3.06
2	3.04	2.89	3.16	3.06
3	3.09	3.01	3.20	3.05
4	3.21	3.00	3.61	3.03
5	3.18	2.93	3.60	3.01
6	3.44	2.83	4.52	2.97
7	3.48	2.70	4.69	3.05

**TABLE 2.** Contrast Recovery Coefficient and Background Variability

Sphere size (mm)	Contrast recovery coefficient (%)
10	72.9*
13	76.9
17	81.7
22	80.4
28	80.3
37	89.4

* Background variability for 10-mm sphere was 11.3%.

### Phantom Studies

**FIGURE 3.**
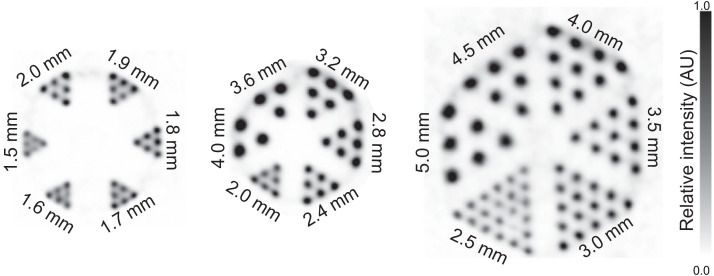
Multilayer Derenzo phantom imaging. Reconstructed images demonstrate hot-rod groups with diameters ranging from 1.5 to 5.0 mm.

Figure 3 shows multilayer Derenzo phantom images acquired at the center of the FOV with TOF-OSEM reconstruction, in which the 1.7-mm hot rods are visually distinguishable. At the center of the FOV with TOF reconstruction, the valley-to-peak ratio (∼0.433) for the 1.7-mm rods is well below the Rayleigh criterion threshold of 0.735, indicating clear rod separation (Supplemental Fig. 5). Without TOF reconstruction, although the signal-to-noise ratio is reduced, the 1.7-mm rods remained visually resolvable at both the central position and at a 5-cm radial offset.

**FIGURE 4.**
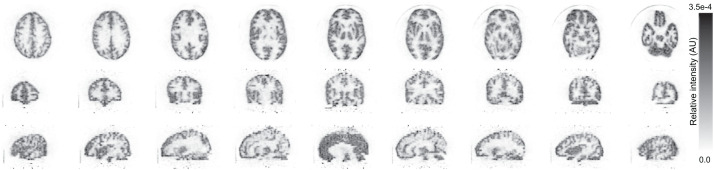
Custom-designed Hoffman brain phantom imaging results. Representative transaxial (top row), coronal (middle row), and sagittal (bottom row) slices reconstructed with TOF-OSEM.

Figure 4 shows reconstructed images of the custom-designed Hoffman brain phantom. Transaxial, coronal, and sagittal slices demonstrated clear visualization of cortical gray matter activity, with distinct separation from nonradioactive white matter and ventricular regions. The images reproduce the complex gyral patterns of the cortex, highlighting the system’s ability to resolve detailed brain anatomy. These findings complement the Derenzo phantom results, confirming both fine spatial resolution and realistic brain structure imaging capability.

### Human Study

**FIGURE 5.**
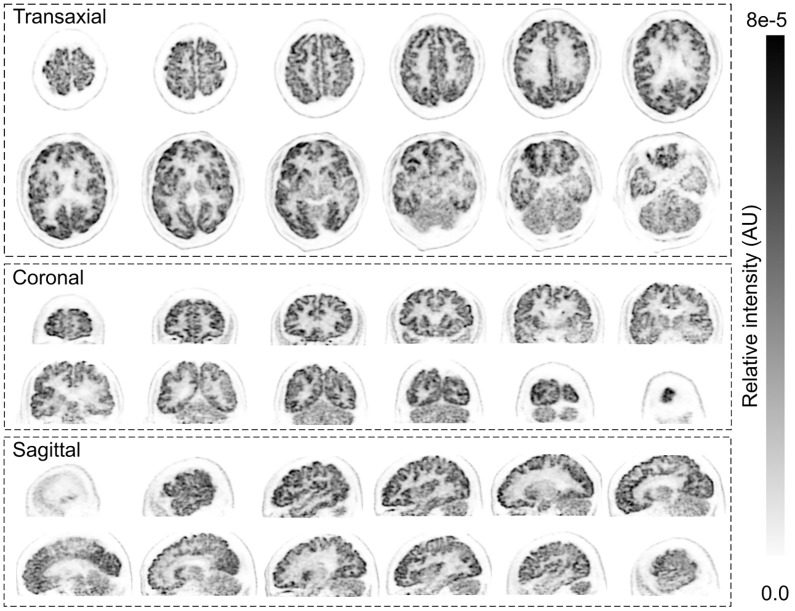
Human brain ^18^F-FDG images acquired with SmartBrain system. Transaxial (top row), coronal (middle row), and sagittal (bottom row) slices show clear cortical uptake and preserved gyral structures.

Figure 5 shows reconstructed human brain images acquired with SmartBrain. Transaxial, coronal, and sagittal views clearly depict cortical uptake patterns, with well-defined gray matter distribution and preserved gyral anatomy. The white matter and ventricular regions exhibited low to no activity, consistent with the expected ^18^F-FDG distribution. These images confirm that SmartBrain can reliably capture fine structural details of the human brain, even in prolonged acquisitions.

**FIGURE 6.**
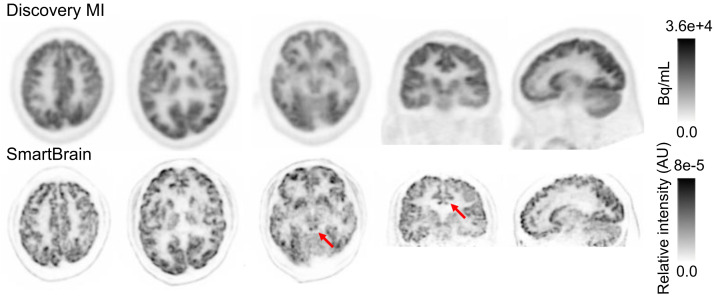
Comparison of human brain ^18^F-FDG images between DMI and SmartBrain systems. Both systems reveal cortical uptake patterns, whereas SmartBrain provides enhanced delineation of gyral details (red arrows). DMI images are displayed in calibrated units (Bq/mL). SmartBrain images were not cross-calibrated; reconstructed voxel values are proportional to activity concentration but expressed in relative units.

Figure 6 compares brain images acquired with the SmartBrain system and the DMI PET/CT scanner. The DMI system produced high-quality images under conventional whole-body PET/CT conditions. SmartBrain yielded similarly detailed cortical uptake patterns, with distinct gyral structures. Notably, SmartBrain demonstrated enhanced visualization of cortical features despite the later imaging time point (∼190 min postinjection, with reduced activity). This comparison highlights SmartBrain’s high spatial resolution, supporting its suitability for brain-focused clinical research.

## DISCUSSION

The SmartBrain system represents one of the few truly wearable brain PET systems capable of imaging under free-moving conditions. It demonstrated excellent spatial resolution, with point-source measurements at the center of 2.29 mm and clear resolution of 1.7 mm rods in the multilayer Derenzo phantom, meeting the requirements for human brain imaging. In the human study, although the sensitivity of the wearable system is lower than the DMI system (14), a 60-min scan acquired after low-dose ^18^F-FDG injection provided clear delineation of brain structures (Fig. 5), with good comfort and feasibility during wear. Furthermore, after applying delay calibration algorithms, the system achieved a coincidence timing resolution of 234 ps. This improved timing performance enhanced TOF-based localization and noise suppression, thereby improving image quality under low-activity conditions. At standard injected activity levels, the same timing performance is expected to further support improved image quality or reduced scan durations.

Several design features contribute to SmartBrain’s performance. First, its full-ring configuration ensures complete FOV coverage, with spatial resolution maintained across central and off-center regions. Second, the use of short 5-mm crystals minimizes DOI uncertainty and reduces Compton scatter within the crystal. Third, the 3-mm crystal pitch further limits intercrystal scatter and supports high-resolution imaging. Fourth, the compact bore size (20 cm) decreases the impact of noncollinearity, which is particularly advantageous in brain imaging. Finally, the incorporation of TOF reconstruction significantly improves image quality and compensates for reduced sensitivity relative to large clinical PET/CT systems (15,16). Together, these elements explain why SmartBrain achieves high spatial resolution and clear visualization of brain structures despite its compact and lightweight design.

Compared with conventional brain PET systems (17,18), SmartBrain offers unique advantages in mobility, cost, and user comfort. Recent efforts toward portable or helmet-based brain PET systems have demonstrated the feasibility of on-head imaging and improved accessibility (19,20); however, these systems remain limited by system weight, geometric flexibility, or imaging performance. In parallel, high-sensitivity brain-dedicated PET systems, such as VRAIN (21) and NeuroEXPLORER (22), achieve excellent image quality and sensitivity but are large, stationary installations that do not support wearable or task-based imaging. In contrast, SmartBrain’s compact detector design, lightweight electronics, and optimized mechanical structure significantly reduce system weight and cost, enabling wearable use. Compared with previous wearable PET prototypes, such as AM-PET (23) and MindTracker (6), SmartBrain shows improvements in spatial resolution and axial coverage and supports dynamic imaging under naturalistic conditions. Design elements such as flexible padding and ergonomic support help minimize motion-related artifacts during wear.

Nonetheless, current limitations remain. The system’s thinner crystals result in lower sensitivity, and although TOF has been implemented, further improvements in detector coverage and sensitivity are needed. In addition, the absence of pile-up correction constrains performance under higher count-rate conditions, and fast dynamic studies remain challenging. Future optimization—including enhanced detector geometry, advanced correction algorithms, and artificial intelligence–assisted image reconstruction (24–26) will further improve image quality, reduce acquisition times, and expand the scope of clinical and neuroscientific applications.

## CONCLUSION

SmartBrain demonstrated brain-level spatial resolution and dynamic imaging capabilities under free-moving conditions. The system’s lightweight, compact design and wearable configuration offer new opportunities for brain PET imaging beyond the constraints of conventional static systems. The system enables ambulatory imaging with enhanced cost-efficiency and a space-saving design, positioning it as a scalable solution for precision diagnostics in both clinical and community health care settings.

## DISCLOSURE

This work was supported by grants from the National Natural Science Foundation of China (82327809) and National Key Research and Development Program of China (2024YFF0727400 and 2024YFF0507700) and by Shenzhen Bay Laboratory High Performance Computing and Informatics Core. This study was approved by the Medical Ethics Committee of the Seventh Affiliated Hospital of Sun Yat-sen University (Approval No. KY-2024-354-02). No other potential conflict of interest relevant to this article was reported.

## Notes

KEY POINTS **QUESTION:** Does the SmartBrain system achieve performance metrics comparable to those of commercial PET systems when evaluated against the NEMA NU 2-2018 standard? **PERTINENT FINDINGS:** SmartBrain provides a spatial resolution below 1.7 mm, a sensitivity of 720.2 cps/MBq, and a timing resolution of 234 ps. **IMPLICATIONS FOR PATIENT CARE:** SmartBrain enables ambulatory imaging with enhanced cost-efficiency and a space-saving design, positioning it as a scalable solution for precision diagnostics in both clinical and community health care settings.

## References

[1] Miyazaki T Nakajima W Hatano M. Visualization of AMPA receptors in living human brain with positron emission tomography. Nat Med. 2020;26:281–288. 31959988 10.1038/s41591-019-0723-9

[2] Van Dyck CH Swanson CJ Aisen P. Lecanemab in early Alzheimer’s disease. N Engl J Med. 2023;388:9–21. 36449413 10.1056/NEJMoa2212948

[3] Yamamoto S Honda M Oohashi T. Development of a brain PET system, PET-Hat: a wearable PET system for brain research. IEEE Trans Nucl Sci. 2011;58:668–673.

[4] Tashima H Yoshida E Nishikido F. Development of the helmet-chin PET prototype. Proceedings of the IEEE Nuclear Science Symposium and Medical Imaging Conference (NSS/MIC). Institute of Electrical and Electronics Engineers; 2015:1155.

[5] Majewski S Proffitt J Brefczynski-Lewis J. HelmetPET: a silicon photomultiplier based wearable brain imager. 2011 IEEE Nuclear Science Symposium Conference Record. Institute of Electrical and Electronics Engineers; 2011:4030–4034.

[6] Xu J Zhao Z Xie S. Mind-tracker PET: a wearable PET camera for brain imaging. Paper presented at *IEEE* Nuclear Science Symposium (NSS) and Medical Imaging Conference (NSS/MIC). October 2017; Atlanta, GA.

[7] Majewski S. The path to the “ideal” brain PET imager: the race is on, the role for TOF PET. Nuovo Cimento C. 2020;43:1–35.

[8] Yu X Liu H Zhao H. A multiplexing method based on multidimensional readout method. Phys Med Biol. 2025;70:045016. 10.1088/1361-6560/adae4c 39854836

[9] Tao W Weng F Chen G. Design study of fully wearable high-performance brain PETs for neuroimaging in free movement. Phys Med Biol. 2020;65:135006. 32325449 10.1088/1361-6560/ab8c90

[10] NEMA Standards Publication NU 2-2018: Performance Measurements of Positron Emission Tomographs. National Electric Manufacturers Association; 2018.

[11] NEMA Standards Publication NU 4-2008: Performance Measurements of Small Animal Positron Emission Tomographs. Rosslyn, VA: National Electrical Manufacturers Association; 2008.

[12] NEMA Standards Publication NU 2-2012: Performance Measurements of Positron Emission Tomographs. Rosslyn, VA: National Electrical Manufacturers Association. 2012.

[13] Gong K Cherry SR Qi J. On the assessment of spatial resolution of PET systems with iterative image reconstruction. Phys Med Biol. 2020;65:225001. 26864088 10.1088/0031-9155/61/5/N193 PMC4890626

[14] Pan T Einstein SA Kappadath SC. Performance evaluation of the 5‐ring GE Discovery MI PET/CT system using the National Electrical Manufacturers Association NU 2‐2012 Standard. Med Phys. 2019;46:3025–3033. 31069816 10.1002/mp.13576 PMC7251507

[15] Surti S Karp JS. Update on latest advances in time-of-flight PET. Phys Med. 2020;80:251–258. 33212421 10.1016/j.ejmp.2020.10.031 PMC7749844

[16] Gonzalez-Montoro A Pavón N Barberá J. Design and proof of concept of a double-panel TOF-PET system. EJNMMI Phys. 2024;11:73. 39174856 10.1186/s40658-024-00674-8 PMC11341523

[17] Catana C. Development of dedicated brain PET imaging devices: recent advances and future perspectives. J Nucl Med. 2019;60:1044–1052. 31028166 10.2967/jnumed.118.217901 PMC6681695

[18] Moliner L Rodríguez-Alvarez MJ Catret JV. NEMA performance evaluation of CareMiBrain dedicated brain PET and comparison with the whole-body and dedicated brain PET systems. Sci Rep. 2019;9:15484. 31664096 10.1038/s41598-019-51898-z PMC6820763

[19] Bartlett EA Lesanpezeshki M Anishchenko S. Dynamic human brain imaging with a portable PET camera: comparison to a standard scanner. J Nucl Med. 2024;65:320–326. 38124218 10.2967/jnumed.122.265309 PMC10858383

[20] Zhou F D’Ascenzo N Zhang B. Development and evaluation of a portable MVT-based all-digital helmet PET scanner. IEEE Trans Radiat Plasma Med Sci. 2024;8:287–294.

[21] Akamatsu G Takahashi M Tashima H. Performance evaluation of VRAIN: a brain-dedicated PET with a hemispherical detector arrangement. Phys Med Biol. 2022;67:225011. 10.1088/1361-6560/ac9e87 36317319

[22] Li H Badawi RD Cherry SR. Performance characteristics of the NeuroEXPLORER, a next-generation human brain PET/CT imager. J Nucl Med. 2024;65:1320–1326. 38871391 10.2967/jnumed.124.267767 PMC11294061

[23] Bauer CE Brefczynski‐Lewis J Marano G. Concept of an upright wearable positron emission tomography imager in humans. Brain Behav. 2016;6:e00530. 27688946 10.1002/brb3.530 PMC5036439

[24] Hu R Li C Tian K. Deep unrolled primal dual network for TOF-PET list-mode image reconstruction. Phys Med Biol. 2025;70. 10.1088/1361-6560/adf9b7 40780257

[25] Schramm G Holler M. Fast and memory-efficient reconstruction of sparse Poisson data in listmode with non-smooth priors with application to time-of-flight PET. Phys Med Biol. 2022;67. 10.1088/1361-6560/ac71f1 PMC9361154 35594853

[26] Murat H Zulkifli MAA Said MA. Optimizing time-of-flight of PET/CT image quality via penalty β value in Bayesian penalized likelihood reconstruction algorithm. Radiography (Lond). 2025;31:343–349. 39733504 10.1016/j.radi.2024.12.011

